# Discovery in the Necropolis of Thebes

**Published:** 1891-11

**Authors:** 


### DISCOVERY IN THE NECROPOLIS OF THEBES.

On February 6 a discovery was made in the necropolis of Thebes second only in importance to the discovery of the royal mummies at Dehr-el-Bahari by M. Maspero, in 1881. About half a mile from Dehr-el-Bahari, a pit has been found containing several hundred magnificent mummies. These, like the royal mummies, had evidently been removed from the tomb and concealed in this receptacle, as a precaution, by the servants of the priests, probably at the same time and for the same reason which caused the royal mummies to be placed in the receptacle where they were found by M. Maspero. This removal is believed by M. Maspero to have taken place in the reign of Aauputh, son of Shasang, of the twenty-second dynasty (*circa*) 966 B. C.

The coffins hitherto found all belong to the twenty-first dynasty and are those of the priests of Ra-Amun and their families. The pit is about forty-five feet in depth, at the bottom of which are two corridors filled with coffins and treasures of every description. In the lower corner—which as yet has only been explored—it is computed that there are some two hundred coffins, and the second corridor is believed to be not less extensive.

The shaft is forty-five feet deep, its mouth is about twelve feet in diameter, and its sides are of rough limestone. One of M. Grebaut's native assistants, who was superintending the work of hauling up the mummy cases, told me that he had been the first actually to enter the corridor where the mummies and treasures lie. The shaft had then been execavated only as deep as the mouth of the corridor, and he crept in on his hands and knees and stood on what he describes as being like a place of enchantment.

The corridor, he said, is some ten or twelve feet high and 250 feet long. It runs in a northerly direction from the shaft toward the Theban hill. At the end there is a short corrider branching from it at right angles, and at some height above the floor at the end is the entrance to a second very long corridor full of treasures, which has been sealed up for the present by M. Grebaut.

Groups of mummies are placed at intervals in families. The number in each group varies from two to six or seven—father, mother and children—and around them exquisitely arranged, are vases, models of houses, models of dahabiehs, cases and boxes full of ushabits, statuettes and every conceivable treasure of ancient Egypt. Without even a speck of dust upon them, this profusion of treasures had remained unlooked at by any eye for nearly 3,000 years. He said that photographs had been taken of the place in its undisturbed state, which he declared to be that of a perfectly kept and well arranged museum.

